# Recipient and Donor Outcomes After Living-Donor Liver Transplant for Unresectable Colorectal Liver Metastases

**DOI:** 10.1001/jamasurg.2022.0300

**Published:** 2022-03-30

**Authors:** Roberto Hernandez-Alejandro, Luis I. Ruffolo, Kazunari Sasaki, Koji Tomiyama, Mark S. Orloff, Karen Pineda-Solis, Amit Nair, Jennie Errigo, M. Katherine Dokus, Mark Cattral, Ian D. McGilvray, Anand Ghanekar, Steven Gallinger, Nazia Selzner, Marco P. A. W. Claasen, Ron Burkes, Koji Hashimoto, Masato Fujiki, Cristiano Quintini, Bassam N. Estfan, Choon Hyuck David Kwon, K. V. Narayanan Menon, Federico Aucejo, Gonzalo Sapisochin

**Affiliations:** 1 Division of Transplantation and Hepatobiliary Surgery, Department of Surgery, University of Rochester Medical Center, Rochester, New York; 2 Department of Surgery, Digestive Disease Institute, Cleveland Clinic, Cleveland, Ohio; 3 HPB and Multi-Organ Transplant Program, Division of General Surgery, University Health Network, Toronto, Ontario, Canada; 4 Division of HPB and Transplant Surgery, Department of Surgery, Erasmus MC Transplant Institute, University Medical Centre Rotterdam, Rotterdam, the Netherlands; 5 Department of Gastrointestinal Oncology, Cancer Institute, Cleveland Clinic, Cleveland, Ohio; 6 Department of Gastroenterology and Hepatology, Digestive Disease Institute, Cleveland Clinic, Cleveland, Ohio

## Abstract

**Importance:**

Colorectal cancer is a leading cause of cancer-related death, and nearly 70% of patients with this cancer have unresectable colorectal cancer liver metastases (CRLMs). Compared with chemotherapy, liver transplant has been reported to improve survival in patients with CRLMs, but in North America, liver allograft shortages make the use of deceased-donor allografts for this indication problematic.

**Objective:**

To examine survival outcomes of living-donor liver transplant (LDLT) for unresectable, liver-confined CRLMs.

**Design, Setting, and Participants:**

This prospective cohort study included patients at 3 North American liver transplant centers with established LDLT programs, 2 in the US and 1 in Canada. Patients with liver-confined, unresectable CRLMs who had demonstrated sustained disease control on oncologic therapy met the inclusion criteria for LDLT. Patients included in this study underwent an LDLT between July 2017 and October 2020 and were followed up until May 1, 2021.

**Exposures:**

Living-donor liver transplant.

**Main Outcomes and Measures:**

Perioperative morbidity and mortality of treated patients and donors, assessed by univariate statistics, and 1.5-year Kaplan-Meier estimates of recurrence-free and overall survival for transplant recipients.

**Results:**

Of 91 evaluated patients, 10 (11%) underwent LDLT (6 [60%] male; median age, 45 years [range, 35-58 years]). Among the 10 living donors, 7 (70%) were male, and the median age was 40.5 years (range, 27-50 years). Kaplan-Meier estimates for recurrence-free and overall survival at 1.5 years after LDLT were 62% and 100%, respectively. Perioperative morbidity for both donors and recipients was consistent with established standards (Clavien-Dindo complications among recipients: 3 [10%] had none, 3 [30%] had grade II, and 4 [40%] had grade III; donors: 5 [50%] had none, 4 [40%] had grade I, and 1 had grade III).

**Conclusions and Relevance:**

This study’s findings of recurrence-free and overall survival rates suggest that select patients with unresectable, liver-confined CRLMs may benefit from total hepatectomy and LDLT.

## Key Points

**Question:**

What are the estimated overall and recurrence-free survival outcomes after living-donor liver transplant (LDLT) in patients with liver-confined, unresectable colorectal cancer liver metastasis (CRLM)?

**Findings:**

In this cohort study of 10 adults with CRLM who received LDLT, Kaplan-Meier estimates of recurrence-free and overall survival at a median follow-up of 1.5 years were 62% and 100%, respectively. Perioperative outcomes for both recipients and donors were consistent with established benchmarks.

**Meaning:**

The results suggest that LDLT may be a viable treatment option for select patients with unresectable CRLMs with favorable tumor biology.

## Teaser

This cohort study examines overall and recurrence-free survival outcomes in adult patients with liver-confined, unresectable colorectal cancer liver metastases who underwent a living-donor liver transplant.

## Introduction

Colorectal cancer is the third most common cancer worldwide, and more than 50% of patients with colorectal cancer develop colorectal cancer liver metastases (CRLMs).^1,2^ Most patients with liver-confined CRLMs are not able to undergo curative hepatectomy owing to multiple bilobar tumors and an insufficient future liver remnant.^3,4,5^ Thus, new strategies that address liver metastases in patients with unresectable CRLMs are needed. Compared with chemotherapy, liver transplant has been reported to provide durable long-term survival in patients with liver-confined CRLMs.^6^ However, the short supply of liver allografts limits the feasibility of this approach in regions with critical shortages.^7^

In the US, liver allograft shortages are common, and approximately 1 in 6 patients awaiting a liver transplant dies every year.^8^ Furthermore, recent changes to the allocation system in the US have shifted the available deceased-donor liver transplant (DDLT) grafts toward patients with higher Model of End-stage Liver Disease (MELD) scores and away from patients with liver tumors.^9^ Thus, alternative strategies to provide liver grafts for patients with unresectable CRLMs are necessary. Outcomes after living-donor liver transplant (LDLT) have been shown to be comparable with those after DDLT without affecting the DDLT allograft pool.^10^ Furthermore, recent reports also suggest that patients who receive an LDLT for hepatocellular carcinoma have a survival advantage compared with those receiving a DDLT.^11^

In this article, we report the first cohort study, to our knowledge, to use LDLT for unresectable, liver-confined CRLMs from 3 high-volume North American centers experienced in hepatobiliary oncology and LDLT.

## Methods

In this cohort study, independent treatment protocols were established at the University of Rochester Medical Center (Rochester, New York) (NCT05248581), the Cleveland Clinic (Cleveland, Ohio), and the University Health Network (Toronto, Ontario, Canada) (NCT02864485). The 3 protocols adhered to the International Hepato-Pancreato-Biliary Association Consensus Guidelines on liver transplant for nonresectable CRLMs.^12^ Prospective registries of patients treated with these protocols were approved by the respective institutional review boards. Inclusion and exclusion criteria for each center’s protocol are available in the eTable in the Supplement. Of note, protocols required all patients to undergo cross-sectional imaging with computed tomography or magnetic resonance imaging along with positron emission tomography to assess for tumor response prior to LDLT. Patients with progression of disease receiving systemic treatment were not eligible for transplant. This study was approved by the institutional review boards of the Cleveland Clinic Foundation, University of Rochester Medical Center, and University Health Network. Oral and written informed consent was obtained from donors and participants. This study followed the Strengthening the Reporting of Observational Studies in Epidemiology (STROBE) reporting guideline.

Patients who were seen at 1 of the 3 liver transplant centers were evaluated at multidisciplinary conferences that included liver transplant surgeons, hepatobiliary surgeons, medical oncologists, hepatologists, and radiation oncologists. Patients deemed to have liver-confined, unresectable CRLMs were prospectively enrolled into treatment protocols from July 2017 to October 2020. The first patient was enrolled into the prospective registry in December 2017. Patients included in this study underwent an LDLT between December 2017 and May 2021. Candidate liver donors were evaluated, and if deemed fit for donation, patients and donors were educated on the natural history of surgical treatment of CRLMs, including the expected high probability of extrahepatic recurrence as described by the Secondary Cancer (SECA) I and II experience,^13,14^ and the risks to both donor and recipient. After providing informed consent, donors and recipients underwent surgery in a staged fashion to facilitate abdominal exploration of the recipient before the commencement of the donor operation.

Donors were monitored according to institutional standards and were followed up postoperatively for complications associated with the procedure. Recipients were followed up according to institutional standards, which included surveillance cross-sectional imaging and serum tumor marker analysis every 3 months after LDLT for the first year and then every 6 months until 5 years postoperatively at the University of Rochester and the Cleveland Clinic Foundation. At the University Health Network, institutional standards were for patients to be surveilled with tumor marker analysis and cross-sectional imaging every 3 months for 2 years, followed by every 6 months until 5 years postoperatively. The last date of follow-up for this study was May 1, 2021.

### Statistical Analysis

Univariate statistics and Kaplan-Meier estimates were calculated using SAS JMP Pro software, version 13 (SAS Institute). Continuous variables are reported as medians and ranges and categorical variables as the count and percentage of the patient population.

## Results

Through the last follow-up date of May 1, 2021, 91 patients were seen in our institutions for consultation for inclusion into the transplant oncology protocols for unresectable, liver-confined CRLMs. Of these 91 patients, 12 (13%) demonstrated sustained disease control on systemic and/or local therapies and were candidates for transplant. Two patients with high MELD scores received DDLT at a single center. The remaining 10 consecutive patients (11%) met all prerequisites for undergoing LDLT. Demographic and clinicopathologic characteristics of patients who underwent LDLT are detailed in Table 1. Six of the patients in this cohort were male and 4 patients were female; the median age was 45 years (range, 35-58 years), and the median body mass index, calculated as weight in kilograms divided by height in meters squared, was 24.5 (range, 18.9-34.6).

**Table 1.** Clinical, Demographic, and Oncologic Characteristics of Patients With Unresectable CRLMs Who Underwent Total Hepatectomy and Living-Donor LT

Characteristic	Patients (N = 10)^a^
Age, median (range), y	45 (35-58)
Sex	
Male	6 (60)
Female	4 (40)
BMI, median (range)^b^	24.5 (18.9-34.6)
Primary T stage	
T3	6 (60)
T4b	4 (40)
Primary node positive	7 (70)
Tumor grade	
Well differentiated	1 (10)
Moderately differentiated	5 (50)
Poorly differentiated	3 (30)
Not assessed	1 (10)
Lymphovascular invasion	2 (20)
Perineural invasion	1 (10)
Synchronous CRLM	
Yes	9 (90)
No	1 (10)
Primary location	
Right colon	2 (20)
Left colon	4 (40)
Rectum	4 (40)
Chemotherapy cycles, median (range), No.	22.5 (6-37)
History	
Prior liver resection	4 (40)
Hepatic artery infusion chemotherapy	3 (30)
Tumor ablation	3 (30)
Positive tumor gene variation status	
* KRAS*	3 (30)
* TP53*	1 (10)
* SMAD4*	1 (10)
* BRAF*	1 (10)
Clinical Risk Score, median (range)	2.5 (1-4)
Oslo Score, median (range)	1.5 (0-2)
CEA level at time of LT, median (range), ng/mL	7.7 (1.6-56.4)
Time from CRLM diagnosis to LT, median (range), y	1.7 (1.1-7.8)
MELD-Na, median (range)	6 (6-20)
Maximum tumor diameter, median (range), cm	3.85 (1.4-5.9)
Distribution of CRLMs	
Unilobar	2 (20)
Bilobar	8 (80)
Radiographic or chemical response to treatment	10 (100)

Abbreviations: BMI, body mass index; CEA, carcinoembryonic antigen; CRLMs, colorectal liver metastases; LT, liver transplant; MELD-Na, Model of End-stage Liver Disease incorporating sodium levels.

SI Conversion factor: To convert CEA to micrograms per liter, multiply by 1.0.

^a^ Data are presented as the number (percentage) of patients unless otherwise indicated.

^b^ Calculated as weight in kilograms divided by height in meters squared.

Most patients (9) had synchronous CRLMs at the time of colorectal cancer diagnosis; the sole patient with metachronous disease developed a CRLM 16 months after the primary tumor was diagnosed. Of note, 8 patients had primary tumors arising in the left colon (4 patients) or rectum (4 patients). From a pathologic staging perspective, all patients had primary tumors greater than stage T2 (6 T3; 4 T4b). Lymphovascular invasion was present in 2 patients (20%) and perineural invasion in 1 patient. Six patients (60%) had well or moderately differentiated tumors and 3 had poorly differentiated tumors; 1 patient did not have a pathologic assessment of tumor differentiation (Table 1).

The 10 patients who underwent LDLT for CRLMs had undergone extensive oncologic treatment before LDLT, as summarized in Table 2. The median time from diagnosis of CRLMs to LT was 1.7 years (range, 1.1-7.8 years). During this time, 4 patients (40%) underwent liver resection, 3 (30%) underwent hepatic artery infusion chemotherapy, and 3 (30%) underwent tumor ablation (Table 2). The median number of modern chemotherapy cycles before LT was 22.5 (range, 6-37). Of note, all 10 patients exhibited sustained radiographic or chemical (carcinoembryonic antigen) response to pretransplant treatment, and the median serum carcinoembryonic antigen level at the time of LT was 7.7 ng/mL (range, 1.6-56.4 ng/mL) (to convert to μg/L, multiply by 1.0) (Table 1).

**Table 2.** Oncologic Treatment Characteristics of Patients Who Underwent Total Hepatectomy and Living-Donor LT

Patient	Timing of CRLM	Systemic treatment	Prior resection	Local therapy	Time from diagnosis of CRLM to LT, y
1	Synchronous metastases	FOLFOX, FOLFIRI, targeted agent	None	None	1.6
2	Synchronous metastases	FOLFOX, FOLFIRI, targeted agent	None	None	5.5
3	Synchronous metastases	FOLFOX, FOLFIRI, targeted agent	Wedge resection, aborted ALPPS	None	1.6
4	Synchronous metastases	FOLFOX, FOLFIRI, targeted agent	None	None	1.4
5	Synchronous metastases	FOLFOX, targeted agent	Right hemihepatectomy	Ablation	1.1
6	Synchronous metastases	FOLFOXIRI, targeted agent	Bisegmentectomy	Hepatic artery infusion	1.4
7	Synchronous metastases	FOLFOX, FOLFIRI, targeted agent	None	Ablation	2.3
8	Metachronous metastases	FOLFIRI, targeted agent	Right posterior sectionectomy, wedge resection	Ablation, hepatic artery infusion	7.8
9	Synchronous metastases	FOLFIRI, targeted agent	None	None	1.7
10	Synchronous metastases	FOLFIRI, targeted agent	None	Hepatic artery infusion	2.0

Abbreviations: ALPPS, associating liver partition with portal vein ligation for staged hepatectomy; CRLM, colorectal liver metastasis; FOLFIRI, fluorouracil + irinotecan; FOLFOX, fluorouracil + oxaliplatin; FOLFOXIRI, fluorouracil + oxaliplatin + irinotecan; LT, liver transplant.

Patients treated with LDLT exhibited a median Clinical Risk Score of 2.5 (range, 1-4) and a median Oslo Score of 1.5 (range, 0-2), with higher scores indicating a higher rate of recurrence.^15,16,17^ At the time of LDLT, 8 patients exhibited bilobar disease on preoperative imaging, and the remaining 2 patients had a history of right-sided resections with recurrence in the liver remnant. Nine patients exhibited normal liver function (median MELD-sodium score of 6 [range, 6-20]); however, 1 patient had liver dysfunction secondary to extensive hepatic artery infusion therapy. Analysis of tumor gene mutations demonstrated that 3 patients had *KRAS* variations, and variations in *TP53*, *SMAD4*, and *BRAF* were each present in a single patient, respectively (Table 1). The single *BRAF* variation was a loss-of-kinase-activity variation (*BRAF* D594G), as opposed to the well-described *BRAF* V600E, which is associated with constitutive kinase activity.^18^ Of note, this point variation has been described to confer a tumor phenotype similar to that of *BRAF* wild-type colorectal cancer.^19^

The 10 patients who met criteria for LDLT underwent total hepatectomy and received allografts from direct living donors, 8 with right hemigrafts and 2 with left hemigrafts. Grafts ranged from 500 to 1295 cm^3^ in volume with a median volume of 953 cm^3^, and the median cold ischemia time was 123 minutes (range, 42-180 minutes) (Table 3).

**Table 3.** Living-Donor and Graft Characteristics of Patients With Unresectable CRLMs Who Underwent Total Hepatectomy and Living-Donor Liver Transplantation

Characteristic	Outcome (N = 10)
Graft–recipient weight ratio, median (range), %	1.30 (0.82-1.60)
Graft volume, median (range), cm^3^	953 (500-1295)
Cold ischemia time, median (range), min	123 (42-180)
Donor	
BMI, median (range)	25.6 (23.0-39.7)
Sex, No. (%)	
Male	7 (70)
Female	3 (30)
Age, median (range), y	40.5 (27-50)
Length of hospital stay, median (range), d	6 (4-7)
CD complications, No. (%)	
None	5 (50)
I	4 (40)
IIIB	1 (10)

Abbreviations: BMI, body mass index (calculated as weight in kilograms divided by height in meters squared); CD, Clavien-Dindo; CRLMs, colorectal liver metastases.

Postoperatively, 3 recipients experienced no complications, whereas 7 experienced Clavien-Dindo complications of grade II (3 recipients), grade IIIA (2), and grade IIIB (2), including biliary complications, acute rejection, ileus, organ space infection, and hepatic artery thrombosis requiring a return to the operating room to declot the hepatic artery with successful revascularization (Table 4). On pathologic review of the liver specimens, 9 demonstrated active viable tumors and 5 exhibited background liver pathology including cirrhosis, steatosis, and liver scarring and fibrosis (Table 4). Immunosuppression was managed according to institutional protocols and incorporated induction with tacrolimus, steroids, and basiliximab followed by a transition to maintenance immunosuppression with 1 of the mammalian target of rapamycin inhibitors (everolimus or sirolimus). Transition to mammalian target of rapamycin inhibitors occurred approximately 6 months after LDLT according to the 3 institutional protocols.

**Table 4.** Liver Explant Pathology and Postoperative Complications of Patients With Unresectable CRLMs Who Underwent Total Hepatectomy and Living-Donor Liver Transplant

Pathologic and postoperative outcome	Patients, No. (%) (N = 10)
Viable tumor	
Yes	9 (90)
No	1 (10)
Underlying liver histology	
Normal parenchyma	5 (50)
Cirrhosis	3 (30)
Steatosis	1 (10)
Scarring, necrosis, and vascular changes	1 (10)
Portal nodal involvement	
Negative	9 (90)
Positive	1 (10)
CD complications	
None	3 (30)
II	3 (30)
IIIA	2 (20)
IIIB	2 (20)

Abbreviations: CD, Clavien-Dindo; CRLMs, colorectal liver metastases.

Recurrence-free and overall survival Kaplan-Meier analyses are shown in the Figure. With a median follow-up of 1.5 years (range, 0.4-2.9 years), recurrences were observed in 3 patients; 1 patient had a recurrence within the peritoneum after 121 days and, of note, was found to have positive portal lymph nodes in the hepatectomy specimen, despite no evidence of extrahepatic disease on pretransplant high-resolution triple-phase computed tomography and positron emission tomography–computed tomography. Two other patients had a recurrence after 92 and 199 days, respectively, 1 within the transplanted liver and the other outside of the liver. All 3 patients were treated with palliative chemotherapy, and 1 died of disease after 3 months of treatment. At the time of this writing, the other 2 patients have survived 2 or more years after LDLT without evidence of disease. Recurrence-free and overall survival estimates for the cohort at 1.5 years after LDLT were 62% and 100%, respectively (Figure).

**Figure.**
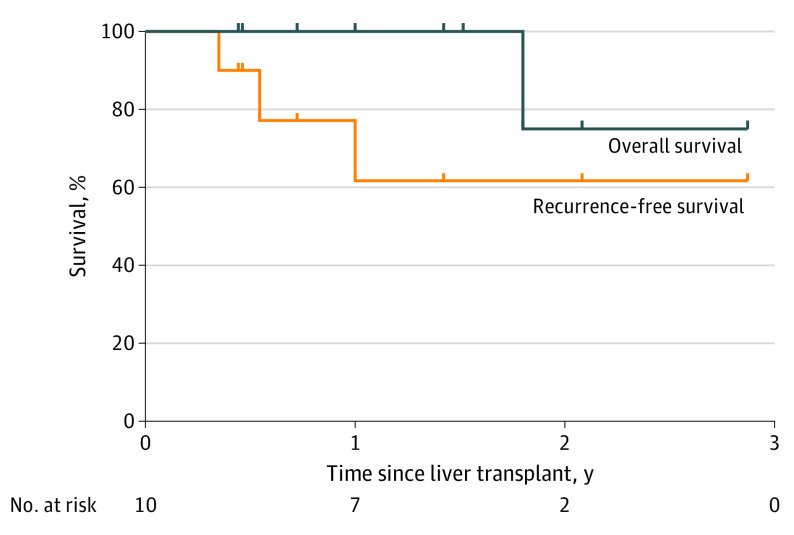
Kaplan-Meier Estimates of Overall and Recurrence-Free Survival in Patients Who Underwent Total Hepatectomy and Living-Donor Liver Transplant

With regard to living-donor outcomes, 7 donors were male, with a median age of 40.5 years (range, 27-50 years) and a median body mass index of 25.6 (range, 23.0-39.7) (Table 3). Intraoperative blood loss was a median of 525 mL (range, 250-1400 mL). All patients were monitored in intensive care or step-down units postoperatively. The median length of hospital stay was 6 days (range, 4-7 days). Five donors experienced no postoperative complications, whereas 4 had Clavien-Dindo grade I complications and 1 experienced a grade IIIB complication owing to a subcutaneous hematoma requiring incision and evacuation. All donors recovered fully postoperatively and were alive and well as of the last follow-up (Table 3).

## Discussion

Liver transplant for CRLMs has emerged as a viable treatment strategy following the reports of Norwegian trials showing the recurrence-free and overall survival rates in highly selected patients.^17^ A comparative surplus of liver allografts in Norway enabled these innovative trials.^13,14^ Adopting this approach, however, will be challenging in most countries owing to the short supply of deceased-donor liver allografts and high rates of waitlist mortality.^8^ Living-donor LT provides an alternative for patients in the US and Canada without further straining the organ-scarce liver waiting list. However, LDLT must be used in clinical scenarios in which the potential benefits for the recipient are carefully weighed against the risk of donor morbidity and mortality.^20^ Selecting patients with unresectable CRLMs who are most likely to have long-term benefit is critical, thus meeting the standard of double equipoise.^17^

Previous experience with LDLT^10^ has demonstrated it is a safe approach for patients with low MELD scores and hepatocellular carcinoma. For unresectable CRLMs, LDLT facilitates the sequencing of treatment. In our experience, the optimal oncologic sequencing for CRLM requires (1) removal of the primary tumor, (2) recovery and additional adjuvant systemic therapy, and (3) potential additional liver-directed therapy. The ability to schedule an LDLT compared with a DDLT thus safely permits the discontinuation of systemic therapy and local therapies before LT, especially for patients with low MELD scores, who otherwise may not be competitive candidates for DDLT.

This study was the first contemporary experience, to our knowledge, to use LDLT to treat patients with unresectable CRLMs. Between December 2017 and May 2021, 10 patients received transplants with living-donor grafts in 3 North American centers. To ensure the highest chance of oncologic success, we selected patients with low Oslo Scores and Clinical Risk Scores who demonstrated sustained response to systemic and local therapies, suggestive of favorable tumor biology. Thus, the median time from diagnosis of CRLM to LT was more than a year. Patients were treated in 3 high-volume liver transplant centers with multidisciplinary teams experienced in both LDLT and hepatobiliary surgery.

With a median follow-up of 1.5 years, the recurrence-free and overall survival in this study’s cohort are consistent with those reported by the SECA II study of highly selected Norwegian patients.^13^ In our study, the estimated overall Kaplan-Meier survival at 1.5 years was 100% and the disease-free survival was 62%. The 3 patients who had recurrence were treated with palliative systemic therapy, and 1 of these patients died of disease. These results were achieved while adequately balancing donor risk and morbidity; all 10 donors were discharged from the hospital 4 to 7 days after surgery and recovered fully.

Our ability to select patients with favorable tumor biology by assessing disease response to systemic therapy may explain, at least partly, the early-term outcomes observed in this cohort. However, future work in understanding the molecular underpinnings of CRLM must enhance risk stratification to better identify a priori which patients may benefit from total hepatectomy and liver transplant. Ongoing work within our institutions comparing the transcriptomic subtype of CRLM tumors that respond to therapy and occur in patients who ultimately undergo LT may provide a novel screening method to identify appropriate candidates more quickly. However, for now, surrogates for disease biology, such as the Oslo Score, the Clinical Risk Score, and sustained clinical response to systemic therapy, remain the key filters through which to select patients who have sufficient opportunity for long-term cancer control, which is necessary to justify the risk to a living donor.

Transplant oncology is a multidisciplinary field that uses liver transplant to replace diseased native livers that have malignant tumors in patients with a good probability of durable oncologic control. With this approach, improved survival has been achieved in patients with hepatocellular carcinoma, hilar cholangiocarcinoma, intrahepatic cholangiocarcinoma, and metastatic neuroendocrine tumors.^21^ In each of these indications, surrogates for tumor biology, such as response to neoadjuvant treatment, tumor size, or number of lesions, have facilitated a balance between the pervasive organ scarcity and acceptable oncologic survival.^21^ In much the same way, new standards are required to enjoin patients with liver-limited CRLMs to national liver waiting lists. Until this occurs, LDLT may represent a critical lifeline for well-selected patients with unresectable, liver-confined CRLMs. From an oncologic perspective, this study’s results are consistent with the experience reported by the Oslo group^13,14^ and reaffirm the capacity of sustained tumor response for select patients who benefit from LT in this setting.

### Limitations

This study has limitations. First, the number of patients included was small, and therefore, conclusions should be made with caution. Second, there was risk of selection bias, given that only patients who received transplants were included in the study. Nonetheless, this study showed that favorable results may be achieved with LDLT in select patients with unresectable CRLMs. The findings should be further investigated in future studies.

## Conclusions

This cohort study found that selected patients with unresectable, liver-confined CRLMs may benefit from total hepatectomy and LDLT, with encouraging rates of recurrence-free and overall survival. Unresectable CRLMs with favorable tumor biology may become an acceptable indication for LT. Careful patient selection remains the key for ensuring acceptable oncologic outcomes for this disease. As more centers begin to use this novel treatment approach, prospective multicenter collaborations must be established to continue to understand and refine the selection and treatment-response criteria. It is time for a North American registry for centers performing LT for unresectable CRLMs. Such a registry will provide a platform for data acquisition in what remains a rare indication for LT, and it may improve understanding of gaps in treatment and of the natural history of posttransplant recurrence and survival. However, LT for CRLMs should be adopted with great caution and only by centers with experienced multidisciplinary teams that include gastrointestinal oncology, transplant oncology, hepatobiliary surgery, and liver transplant. The field of transplant oncology should move toward unified criteria that may facilitate the incorporation of selected patients with CRLMs into the standard organ-allocation systems.

## Notes

Supplement. **eTable.** Inclusion and Exclusion Criteria of Participating LDLT Centers
