# Supplementary material for: Recipient and Donor Outcomes After Living-Donor Liver Transplant for Unresectable Colorectal Liver Metastases
Source: JAMA Surg. 2022 Mar 30;157(6):524–30. doi: 10.1001/jamasurg.2022.0300 (PMC8968681; doi:10.1001/jamasurg.2022.0300)
Supplement: Supplement. — eTable. Inclusion and Exclusion Criteria of Participating LDLT Centers [file jamasurg-e220300-s001.pdf]

Supplementary Online Content

Hernandez-Alejandro R, Ruffolo LI, Sasaki K, et al. Recipient and donor outcomes after living donor liver transplant for unresectable colorectal liver metastases. *JAMA Surg*. Published online March 30, 2022. doi:10.1001/jamasurg.2022.0300

eTable. Inclusion and Exclusion Criteria of Participating LDLT Centers

This supplementary material has been provided by the authors to give readers additional information about their work.

© 2022 Hernandez-Alejandro R et al. *JAMA Surgery*.

eTable. Inclusion and Exclusion Criteria of Participating LDLT Centers

University of Rochester

- Primary Tumor Removed
- Response to Chemotherapy ≥12 months
- No Evidence of Extrahepatic Disease
- CEA <80ng/dL
- Patient ECOG 0 or 1 and age ≤65
- Absence of synergistic tumor mutations (KRAS & TP53)
- Oslo Score ≤ 2

Cleveland Clinic

- > 12 Months From Primary Tumor Removal
- Response to Chemotherapy 6-12months
- No Evidence of Extrahepatic Disease
- CEA <100ng/dL
- Patient ECOG 0 or 1 and age ≤65
- Absence of tumor mutation (BRAF)

University Health Network

- Response to Chemotherapy at least 6 months
- Primary CRC resected > 6 months
- No Evidence of Extrahepatic Disease
- CEA stable or decreasing at all points prior to liver transplant
- Patient ECOG 0 or 1 and age 18-68
- Absence of tumor mutation (BRAF)

© 2022 Hernandez-Alejandro R et al. *JAMA Surgery*.
